# Cranium-Facial Trauma By a Cutting Weapon

**DOI:** 10.1016/S1808-8694(15)30114-2

**Published:** 2015-10-19

**Authors:** Francisco S. Almeida, Paulo R. Pialarissi, José A. Camanducaia, José M. Reis, Natanael J.A. Neves, André Silva

**Affiliations:** a Doctor/USP/SP, staff of the Odontomed Hospital.; b Doctor/USP/SP, Full Professor - Sao Paulo Pontifical Catholic University.; c Neurosurgery specialist, Adjunct Professor of Neurosurgery - Itajuba Medical School.; d Doctor/UFMG/MG, Adjunct Professor of Human Anatomy - Itajuba Medical School.; e Otorhinolaryngology specialist, staff of the Itajuba Saude Ceam Hospital.; f Medical undergraduate student, Itajuba Medical School. Odontomed Hospital.

**Keywords:** foreign body, injure by a cutting weapon, cranium-facial trauma

## INTRODUCTION

Craniofacial trauma caused by metallic objects is common in wars, personal conflict, and a variety of accidents. Causes may include firearms, cutting and thrusting weapons, metal, bone or dental fragments, all of which produce injury ranging from minor abrasions to extensive and severe fractures.

This type of injury occurs mainly in young male adults aged between 19 and 30 years due to increased exposure to predisposing factors.^1^

Penetrating brain injury by low kinetic energy objects is uncommon.^2^ Most cases of craniofacial trauma involve cranial and orbit injury. At other times, the nervous system may be extensively involved, such as in a reported case involving asbestos fiber where the patient underwent craniotomy.^3^ Injury caused by gunshots, which have high kinetic energy, are more extensive and are frequent in major urban centers.^4^ The face is the most affected region in cases of personal violence.

In Brazil 78.31% of homicides result from firearms and 10.96% of homicides result from cutting and thrusting weapons.^5^ Causes of cranial trauma are motor vehicle accidents (40.7%), aggression with or with no weapons (25.4%), and falls (24%).^6^.

## CASE PRESENTATION

ECR, white, male, aged 19 years, was brought in with a penetrating incised wound on the right fronto-orbital suture region caused by a cutting and thrusting weapon (knife) as a result of aggression. The patient was awake, and the general medical, ophthalmological and neurological exams were within normal limits.

**Figure 1:**
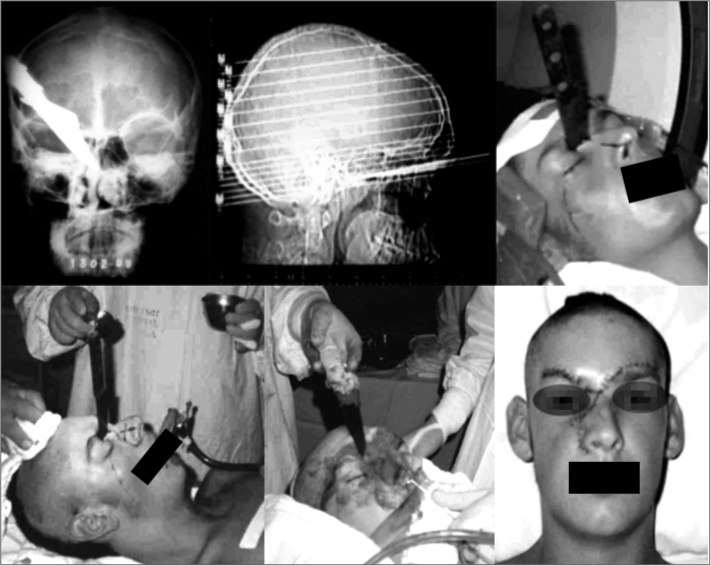
Presentation of the anatomical positions of the foreign body in radiographs and tomography, aspect of the skin, the surgical access, and the patient in the immediate postoperative period.

Imaging revealed a foreign body penetrating the ethmoid bone and reaching the sphenoid sinus (Figure 1).

### Surgical report

An incision was done along the upper rim of the orbit until the right nasogenian sulcus. Three flaps (frontal, right infraorbitary, and left nasogenian) were made and fixed to the skin.

Translocation was done to access the deeper regions, with monoblock removal of bone elements. The first of these was the caudal part of the frontal bone and nasal bones. The second bone element included the medial segments of the lacrimal and maxillary bones. Resection of ethmoid and sphenoid portions was carried out, preserving adjacent structures.

The full penetration length of the foreign body was visualized. This aimed to avoid involvement of important structures upon removal of the foreign body. The surgical would was closed including repositioning of the bone blocks (Figure 1).

The postoperative period was uneventful. At present the patient is in the sixth year of follow-up, with no sequelae.

## DISCUSSION

Iron rods, 2 asbestos fiber, 3 firearms, 4 and knives5 are among the most common weapons used to inflict injury on human beings.

In Brazil facial trauma by cutting and thrusting weapons usually occurs between individuals of lower social and economical level, and are associated with alcohol abuse, violent aggression and robbery.^1^, ^5^, ^6^

A multidisciplinary team is important in the treatment of craniofacial injury to improve the outcome.

Imaging studies are essential to assess the damage caused by foreign bodies to the cranium and the face, to evaluate the extension of injury, and to plan the surgical approach.

Treatment consists of foreign body removal by trauma-free dissection of injured structures, preserving craniofacial function and esthetics.

## FINAL COMMENTS

The authors report an uncommon case of facial trauma by a cutting and thrusting weapon (knife) that penetrated the right fronto-orbital suture region extending to the sphenoid bone.

In such cases, prompt multidisciplinary medical care and careful surgical technique assure a good prognosis for the patient and minimize the risk of postoperative complications.
